# Bacteriocin-Antimicrobial Synergy: A Medical and Food Perspective

**DOI:** 10.3389/fmicb.2017.01205

**Published:** 2017-06-29

**Authors:** Harsh Mathur, Des Field, Mary C. Rea, Paul D. Cotter, Colin Hill, R. Paul Ross

**Affiliations:** ^1^Teagasc Food Research Centre, MooreparkCork, Ireland; ^2^APC Microbiome Institute, University College CorkCork, Ireland; ^3^School of Microbiology, University College CorkCork, Ireland

**Keywords:** bacteriocins, antibiotic resistance, synergy, stressors, pathogens, antimicrobials, combinations

## Abstract

The continuing emergence of multi-drug resistant pathogens has sparked an interest in seeking alternative therapeutic options. Antimicrobial combinatorial therapy is one such avenue. A number of studies have been conducted, involving combinations of bacteriocins with other antimicrobials, to circumvent the development of antimicrobial resistance and/or increase antimicrobial potency. Such bacteriocin-antimicrobial combinations could have tremendous value, in terms of reducing the likelihood of resistance development due to the involvement of two distinct mechanisms of antimicrobial action. Furthermore, antimicrobial synergistic interactions may also have potential financial implications in terms of decreasing the costs of treatment by reducing the concentration of an expensive antimicrobial and utilizing it in combination with an inexpensive one. In addition, combinatorial therapies with bacteriocins can broaden antimicrobial spectra and/or result in a reduction in the concentration of an antibiotic required for effective treatments to the extent that potentially toxic or adverse side effects can be reduced or eliminated. Here, we review studies in which bacteriocins were found to be effective in combination with other antimicrobials, with a view to targeting clinical and/or food-borne pathogens. Furthermore, we discuss some of the bottlenecks which are currently hindering the development of bacteriocins as viable therapeutic options, as well as addressing the need to exercise caution when attempting to predict clinical outcomes of bacteriocin-antimicrobial combinations.

## Introduction

The recent increase in the number of cases of antibiotic resistance has encouraged scientists to reassess alternative therapeutic options (Michael et al., 2014; Holmes et al., 2016). The rate of antibiotic resistance is worrying since progress in the discovery of novel antibiotics with different modes of action has slowed significantly. There are some exceptions, such as the relatively recent discovery of teixobactin, which exhibits activity against Gram positive pathogens including methicillin-resistant *Staphylococcus aureus* (MRSA), *Streptococcus pneumoniae* and mycobacteria, and which has a distinct mechanism of action involving the inhibition of peptidoglycan synthesis (Ling et al., 2015). Encouragingly, teixobactin proved to be effective in mouse trials in decreasing the load of *S. pneumoniae* and MRSA. Furthermore, *S. aureus* and *M. tuberculosis* isolates displaying resistance to teixobactin could not be isolated under laboratory conditions (Ling et al., 2015). In general, however, the widespread discovery of novel antibiotics remains largely uncommon (Cooper and Shlaes, 2011).

One option to compensate for the dearth of novel antibiotics is to introduce bacteriocins as therapeutic options in clinical settings. Bacteriocins are ribosomally-synthesized antimicrobial peptides produced by bacteria and can exhibit narrow spectra of activity (targeting members of the same species), whereas others display broader activity spectra (targeting other species and genera) (Cotter et al., 2013). Bacteriocins are broadly classified into class I (post-translationally modified) and class II (unmodified) groups. The most extensively studied subclass of bacteriocins is the lantibiotics, which includes nisin, lacticin 3147, mersacidin, lacticin 481 and staphylococcin C55, amongst others (Brötz et al., 1995; McAuliffe et al., 1998; Navaratna et al., 1998; Xie et al., 2004; Field et al., 2008). Several lantibiotics exhibit potent activity against clinically relevant and food-borne pathogens (Mota-Meira et al., 2000; Kruszewska et al., 2004; Cotter et al., 2005, 2013; Rea et al., 2007; de Kwaadsteniet et al., 2009; Piper et al., 2009; Jabes et al., 2011; Field et al., 2012, 2015). Significantly, some lantibiotics have been shown to possess activity against antibiotic-resistant targets such as vancomycin-resistant enterococci (VRE) and MRSA (Chatterjee et al., 1992; Kruszewska et al., 2004; Piper et al., 2009). In a number of instances, the receptor for the lantibiotic subclass of bacteriocins is the peptidoglycan precursor lipid II, which is also the binding site for vancomycin, albeit at a different site within the precursor (Breukink and de Kruijff, 2006). The class II bacteriocins, which are unmodified or cyclic in nature, are further divided into class IIa-IIe (Cotter et al., 2013). The class IIa subgroup of bacteriocins generally have strong activity against the food-borne pathogen, *Listeria monocytogenes* (Pucci et al., 1988; Eijsink et al., 1998; Gravesen et al., 2002; Héchard and Sahl, 2002; Dabour et al., 2009). In contrast to class I bacteriocins, several class IIa bacteriocins bind the mannose phosphotransferase receptor (Man-PTS; Oppegård et al., 2007). Overall, bacteriocins exhibit strong activity against their target strains, often in the nanomolar range, rendering them more potent than their antibiotic counterparts in certain cases (Mathur et al., 2013; Ming et al., 2015). Thus, bacteriocins on their own have potential for use in clinical applications.

However, perhaps an even better option is to combine bacteriocins with other existing antibiotics/antimicrobials. It is plausible that using antimicrobials that function synergistically with bacteriocins may expedite each other's killing effects, thereby possibly reducing the likelihood of resistance development to either the bacteriocin or the antimicrobial stressor. Furthermore, combinations of bacteriocins with antibiotics can decrease the concentration of antibiotics required to kill a target pathogen, thereby diminishing the likelihood of adverse side effects associated with the antibiotic. An example of such undesirable effects is the nephrotoxicity associated with the polymyxin group of antibiotics (Mendes et al., 2009; Abdelraouf et al., 2012). Synergistic combinations of bacteriocins with antibiotics can also reduce the financial burden associated with the synthesis and administration of the more expensive antibiotics. Finally, successful synergistic interactions between bacteriocins and other antimicrobials can broaden the spectrum of activity, which may be useful in treating clinical infections of unknown etiology.

A variety of different methods assessing antimicrobial synergy in laboratory conditions have been described in the literature. Examples of such tests include the broth-based checkerboard assay, as well as agar-based screens such as E-tests (bioMérieux) to evaluate synergy (Orhan et al., 2005; Tsuji and Rybak, 2006; Foweraker et al., 2009; Sopirala et al., 2010; Soltani et al., 2012). However, there is a general consensus that agar-based screens are relatively crude compared to the more accurate broth-based methods. The checkerboard method permits the determination of the fractional inhibitory concentration (FIC) index. Although some researchers disagree about the interpretation of results obtained with checkerboard assays, there appear to be five different effects on which the majority of researchers have reached a consensus. These five effects are (i) full synergy (FIC ≤ 0.5), (ii) partial synergy (0.5 ≤ FIC ≤ 0.75), (iii) additive effects (0.75 ≤ FIC ≤ 1.0), (iv) indifferent effects (1.0 ≤ FIC ≤ 2.0) and (v) antagonistic effects (FIC ≥ 2.0) (Bacon et al., 1991; Orhan et al., 2005). The main disadvantage of the checkerboard assay is that it can only examine two antimicrobials at a time. On the other hand, an assay known as the multiple combination bactericidal test (MCBT) has the potential to evaluate combinations of up to four antimicrobials concurrently. While checkerboard assays evaluate different antimicrobial concentrations, MCBT assays only evaluate defined set concentrations of antimicrobials. MCBT assays are based on the premise that 99.9% of the bacterial population is killed after 24 h. Synergy can also be assessed by combining antimicrobials together and conducting time-kill assays, whereby samples are taken at defined time points to evaluate the effects of two or more antimicrobials together (Doern, 2014). Finally, it must be emphasized that there can be variations in terms of the types of interactions obtained between different methods. Variations frequently observed between different methods can be ascribed to slight differences in the end-points used in the assays. It should also be noted that *in vitro* results do not necessarily predict the success of different combinations *in vivo*.

In this review, we outline a selection of studies conducted whereby bacteriocins were combined successfully with other bacteriocins, antibiotics, phage lysins and other antimicrobials/stressors such as naturally-derived plant essential oils, with a view to targeting clinical/veterinary pathogens, pathogens involved in biofilms, and food-borne pathogens.

## Effects of antimicrobial combinations involving bacteriocins against clinical and veterinary pathogens

### Lantibiotics in combination with antibiotics against clinical and veterinary pathogens

The lantibiotics are the most extensively studied group of bacteriocins (Cotter et al., 2013). These bacteriocins are post-translationally modified antimicrobial peptides, characterized by the presence of unusual amino acids, and their structure involves lanthionine and β-methyl lanthionine intramolecular bridges. During the formation of these intramolecular bridges, serine and threonine residues are dehydrated to dehydroalanine and dehydrobutyrine respectively (Cotter et al., 2013). In one study, the most thoroughly investigated lantibiotic, nisin (at concentrations ranging from 1.5 to 16 μg/ml), was combined with the glycolipodepsipeptide ramoplanin (at concentrations ranging from 0.38 to 1.5 μg/ml), both of which target lipid II, and the combination resulted in synergistic interactions against 14 out of 20 MRSA strains assessed (Brumfitt et al., 2002). Resistance to methicillin in *S. aureus* is a significant problem and is mediated by the expression of PBP2a (penicillin binding protein 2a) instead of, or in addition to, the regular PBP (Hackbarth et al., 1995; Stapleton and Taylor, 2002; Chambers and Deleo, 2009). Furthermore, β-lactamase expression is also known to contribute to resistance to methicillin amongst *S. aureus* strains (Montanari et al., 1996), rendering such MRSA strains amongst the most challenging pathogens to target in clinical settings. In contrast to the effective nisin-rampolanin interactions however, the study by Brumfitt et al., showed that a combination of nisin with 3–12 μg/ml chloramphenicol (which inhibits protein synthesis in bacteria) yielded antagonistic effects against these MRSA strains (Brumfitt et al., 2002).

*Enterococcus faecalis* is another nosocomial pathogen and is an etiological agent of endocarditis, urinary tract infections and other systemic infections, and has exhibited resistance to several groups of antibiotics including aminoglycosides, daptomycin, quinolones, macrolides, rifampicin and β-lactams, amongst others (Murray, 1990; Johnston and Jaykus, 2004; Flores-Mireles et al., 2015). A study by Tong et al., demonstrated that the presence of 200 U/ml nisin led to a reduction in minimum inhibitory concentration (MIC) and minimum bactericidal concentration (MBC) values of 18 different antibiotics against *E. faecalis*. The combinations of 200 U/ml nisin with 1–16 μg/ml chloramphenicol or with 2 μg/ml penicillin were especially effective, with statistically significant improvements in MIC values in the presence of nisin. Synergistic interactions between nisin in combination with either penicillin or chloramphenicol against three *E. faecalis* strains were also apparent with checkerboard assays. In addition, transmission electron microscopy (TEM) demonstrated that these combinations were highly effective at destroying *E. faecalis* cells (Tong et al., 2014a). In another study, the lipid II-binding lantibiotic, actagardine, was combined with the antibiotics metronidazole, vancomycin and ramoplanin against several *Clostridium difficile* isolates (Mathur et al., 2013). *C. difficile* is primarily a nosocomial pathogen and is the causative agent of *C. difficile*-associated diarrhea (CDAD), which often occurs due to perturbations of the gut microbiota resulting from broad-spectrum antibiotics (Rea et al., 2010; Leffler and Lamont, 2015). Interestingly, it was revealed that actagardine in combination with ramoplanin behaved in a partial synergistic/additive fashion against 61.5% of the target *C. difficile* strains assessed in the relatively recent study (Mathur et al., 2013). Actagardine-metronidazole and actagardine-vancomycin combinations were also effective with partial synergistic/additive effects obtained against 54% and 38% of *C. difficile* strains respectively (Mathur et al., 2013).

With respect to veterinary pathogens, LeBel et al., reported that nisin behaved in a synergistic manner when combined with the β-lactams amoxicillin, penicillin or ceftiofur, and also when combined with streptomycin or tetracycline against *Streptococcus suis* (LeBel et al., 2013). Although *S. suis* is primarily a porcine pathogen, it has also been described as a zoonotic pathogen with transmission to humans possible (Hughes et al., 2009; Goyette-Desjardins et al., 2014; Huong et al., 2014). The novel lantibiotic, suicin 3908, was also shown to interact in an additive manner with amoxicillin or penicillin against *S. suis* in a recent study (Vaillancourt et al., 2015).

### Sactibiotics and other groups of bacteriocins, in combination with antimicrobials against clinical pathogens

The sactibiotics are a relatively newly-designated group of bacteriocins, characterized by post-translational modifications involving radical S-adenosyl methionine (SAM) methylases and the presence of sulfur to α-carbon linkages in their structure (Arnison et al., 2013; Mathur et al., 2015). In general, the sactibiotics tend to be relatively hydrophobic in nature with characteristic hairpin-shaped structures. The efficacy of the sactibiotic, thuricin CD, was assessed in combination with metronidazole, ramoplanin and vancomycin, with a view to targeting *C. difficile* strains. It was revealed that thuricin CD interacted in a partial synergistic manner when combined with ramoplanin against 31% of the target strains tested (Mathur et al., 2013). In contrast, combinations of thuricin CD-vancomycin and thuricin CD-metronidazole resulted in indifferent effects (1.0 ≤ FIC ≤ 2.0) against the majority of *C. difficile* isolates tested in the study (Mathur et al., 2013). Antimicrobial combination studies have also been conducted with the sactibiotic subtilosin A against *Gardnerella vaginalis*. This opportunistic pathogen is one of the predominant causative agents of bacterial vaginosis (Catlin, 1992). It was determined that subtilosin A exhibited Bliss synergy when combined with clindamycin phosphate, metronidazole, ε-Poly-L-Lysine (polylysine) and lauramide arginine ethyl ester (LAE) against the pathogen (Cavera et al., 2015). Bliss synergy is based on the principle that drug effects are outcomes of probabilistic processes and assumes that drugs act independently in such a manner that neither of them interferes with the other (different sites of action), but each contributes to a common result (Tang et al., 2015). However, only subtilosin A-metronidazole and subtilosin A-clindamycin combinations yielded synergistic effects against *G. vaginalis* when FIC indices were evaluated (Cavera et al., 2015). Subtilosin produced by *Bacillus amyloliquefaciens* was also shown to exhibit synergistic activity with lauric arginate, ε-poly-L-lysine and glycerol monolaurate against *G. vaginalis* in an earlier study (Noll et al., 2012). Finally, a recent study demonstrated that combinations of the class II bacteriocin, durancin 61A and the broad-spectrum antimicrobial reuterin yielded FIC indices of 0.2 against *C. difficile*, indicating highly synergistic activity (Schaefer et al., 2010; Hanchi et al., 2017). Interestingly, durancin 61A combinations with vancomycin were also synergistic against MRSA (*S. aureus* ATCC 700699) with FIC values of 0.3 obtained (Hanchi et al., 2017).

With respect to the treatment of oral pathogens, one study investigated the combination of the bacteriocin PsVP-10, a non-lantibiotic displaying characteristics akin to class II bacteriocins, with the antimicrobials triclosan and chlorhexidine in an effort to target *Streptococcus mutans* and *Streptococcus sobrinus* (Lobos et al., 2009). *S. mutans* is the predominant causative agent of dental caries (Loesche, 1986; Metwalli et al., 2013), while *S. sobrinus* has also been implicated in its causation, albeit to a lesser extent than *S. mutans* (Conrads et al., 2014). Synergistic effects were obtained when PsVP-10 was combined with chlorhexidine, whereas partial synergistic/additive effects were apparent with PsVP-10-triclosan combinations in the study by Lobos et al. (2009). *Candida albicans* is the etiological agent of many opportunistic yeast infections in the oral cavity as well as other parts of the body and it is noteworthy that the class II bacteriocins plantaricin E, F, J, and K have displayed activity against *C. albicans* when used in combination with several different antibiotics (Sharma and Srivastava, 2014). This opportunistic pathogen typically infects immunosuppressed individuals. Indeed, it is frequently implicated in oro-pharyngeal thrush in AIDS patients and can also be a causative agent of systemic infections such as vaginitis (Kim and Sudbery, 2011). Thus, effective combinations of therapeutics to target this pathogen are warranted.

### Effective bacteriocin-antimicrobial combinations against biofilms

**Figure 1:**
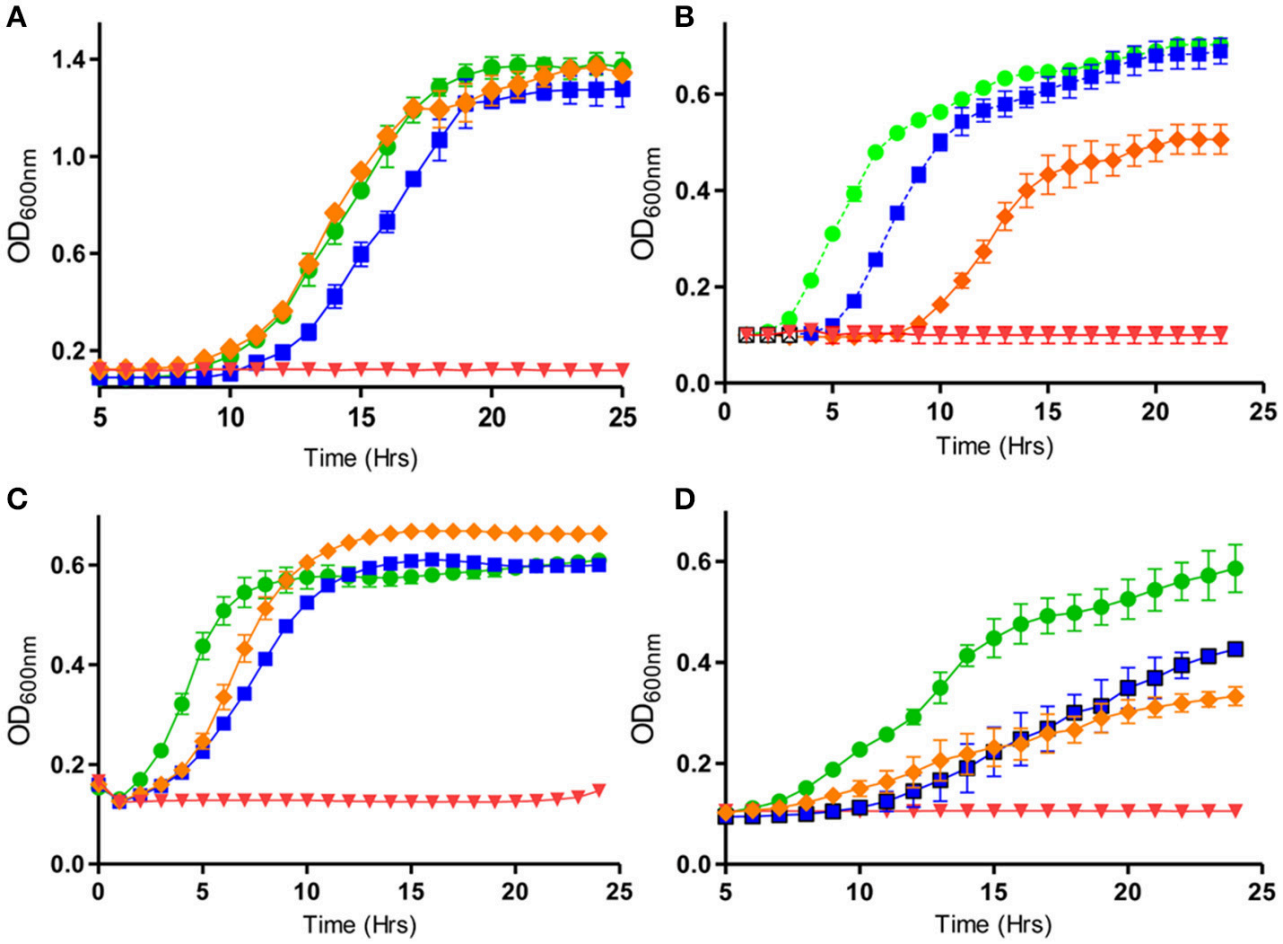
Activity of nisin and bioengineered derivatives thereof, in combination with different antimicrobials against various pathogens: **(A)** *Pseudomonas aeruginosa* PA-01 in the presence of nisin A (50 μg/ml) (orange diamond), colistin (0.75 μg/ml) (blue square), in combination (red diamond) and untreated (green circle), **(B)** *S. pseudintermedius* DSM 21284 in the presence of nisin I4V peptide (0.932 μg/ml) (orange diamond) with penicillin (Pen) (0.8 μg/ml) (blue square), in combination (red diamond), and untreated (green circle). **(C)** *S. aureus* SA113 in the presence of nisin V (3.0 μg/ml), (orange diamond), 1.5 μg/ml chloramphenicol (Cm) (blue square), in combination (red diamond), and untreated (green circle) and **(D)** *E. coli* O157:H7 in the presence of nisin S29A (orange diamond), carvacrol (200 μg/ml) (blue square) and combinations of nisin S29A and carvacrol (red inverted triangle) and untreated (green circle). Adapted from Field et al. (2016a,b) and Campion et al. (2017). Rights and Permissions have been obtained from Copyright Clearance Center's RightsLink service.

**Figure 2:**
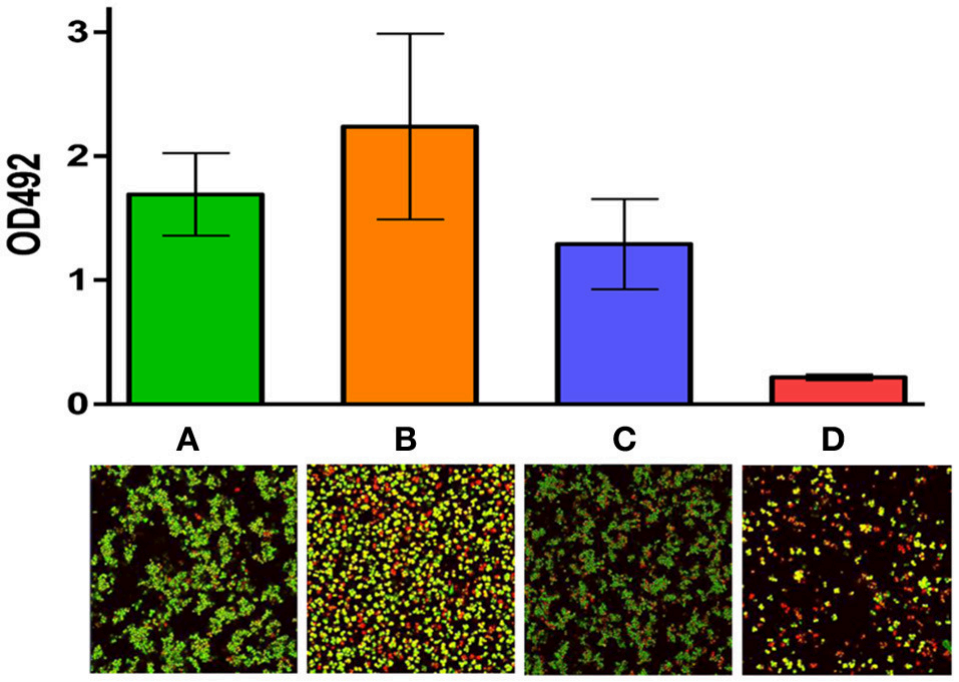
Activity of nisin I4V in combination with chloramphenicol against *S. pseudintermedius* DSM21284 biofilms: Viability of biofilms of *S. pseudintermedius* DSM 21284 when **(A)** untreated, **(B)** treated with 1X MIC chloramphenicol alone, **(C)** treated with 4X MIC nisin I4V peptide alone and **(D)** 1X MIC chloramphenicol and 4X MIC nisin I4V peptide in combination as evaluated by the colorimetric XTT assay and measured using a microtiter plate reader (top) and Live/dead staining confocal images (bottom). Adapted from Field et al. (2016a). Rights and Permissions have been obtained from Copyright Clearance Center's RightsLink service.

Bacteria present in a biofilm are inherently more resistant to antimicrobials than those present in a planktonic state predominantly due to the complex polymeric matrix which biofilms are composed of, and this matrix often impedes penetration of the antimicrobial to the deepest strata of the biofilm (Mah and O'Toole, 2001; Davies, 2003; Flemming and Wingender, 2010). Thus, there is an increased emphasis on trying to seek alternative therapeutic options and/or effective antimicrobial combinations to target biofilms. A recent study showed that nisin interacts synergistically with several antibiotics and such combinations were effective against *Staphylococcus* biofilms (Field et al., 2016a). In particular, the bioengineered variant of nisin, M21V, in combination with penicillin was effective at inhibiting biofilms of *S. aureus* SA113. In contrast to SA113 biofilms however, M21V was most effective in combination with chloramphenicol against SA113 planktonic cells (Figure 1C). Furthermore, another variant, nisin I4V, was reported to be highly effective in conjunction with chloramphenicol against *Staphylococcus pseudintermedius* DSM21284 biofilms in the same study, while combinations of I4V with penicillin were particularly potent against DSM21284 planktonic cells (Field et al., 2016a; Figures 1B, 2). *S. pseudintermedius* is an opportunistic pathogen, primarily affecting dogs (van Duijkeren et al., 2011) and an outbreak of methicillin-resistant *S. pseudintermedius* in Finland has been documented in recent years (Grönthal et al., 2014). Significantly, a case of *S. pseudintermedius* in a human was also described for the first time in 2006 (Van Hoovels et al., 2006). While the use of the colorimetric XTT assay revealed that nisin I4V-chloramphenicol combinations were effective at diminishing the viability of the biofilms of a *S. pseudintermedius* strain in the study by Field and co-workers, the authors found that there was no synergy with nisin-vancomycin combinations against *Staphylococcus* biofilms (Field et al., 2016a). Indeed, variations with respect to the nature of interactions between nisin and vancomycin when targeting MRSA or methicillin-sensitive *S. aureus* (MSSA) have also been reported in previous studies (Dosler and Gerceker, 2011, 2012). A separate study by Mataraci and Dosler also investigated the potency of several antibiotics combined with nisin against MRSA ATCC43300 biofilms and it was determined that antibiotic-nisin combinations were effective at preventing biofilm formation at 1x MIC. However, biofilm-associated bacteria were highly resistant to antibiotics or antibiotic combinations (Mataraci and Dosler, 2012). In another study, it emerged that nisin in combination with ciprofloxacin or daptomycin was effective against 24 h-old MRSA biofilms. The combination elicited a reduction in MRSA CFU counts by 3 logs, whereas the individual antimicrobials were unable to decrease the CFU to such an extent (Dosler and Mataraci, 2013). A recent interesting study evaluated the activity of nisin and lysostaphin against pre-formed biofilms of *S. aureus* involved in bovine mastitis (Ceotto-Vigoder et al., 2016). After treatment for 24 h with a combination of nisin and lysostaphin, the biofilm pre-formed by all eight strains tested in the study was reduced by >50%, as suggested by biofilm detachment from the microtiter plates. Although no remarkable detachment could be noticed by confocal laser scanning microscopy (CLSM) analysis after a 4 h treatment, when the biofilm matrix of *S. aureus* 4181 was assayed for cell viability, most cells were shown to be dead (Ceotto-Vigoder et al., 2016). In contrast to the synergistic activity between nisin and lysostaphin, significantly higher concentrations of lysostaphin used on its own, up to 50-fold higher, were required to cause the same level of biofilm detachment achieved by nisin-lysostaphin combinations. A separate study evaluated the antimicrobial effects of the class IIc bacteriocin enterocin AS-48 both independently and in combination with several biocides against three MRSA and three MSSA strains (Caballero Gómez et al., 2013). It was determined that the anti-biofilm activity of the biocides triclosan, benzalkonium chloride and polyhexamethylene guanidinium chloride (PHMG) were highly effective in combination with 50 μg/ml of AS-48 (Caballero Gómez et al., 2013). Thus, these combinations may prove to be useful therapeutic options for MRSA. Finally, Tong and co-workers also reported the efficacy of nisin in combination with chloramphenicol, ciprofloxacin or penicillin at targeting biofilms of the nosocomial pathogen, *E. faecalis* (Tong et al., 2014a).

With a specific goal of finding therapeutics against oral biofilms, Tong and co-workers also examined the anti-biofilm activities of the lantibiotic nisin, independently and in combination with free amino acids against *S. mutans* biofilms. The results of crystal violet biofilm assays indicated that mixtures of either the L or D-enantiomers of Glu, Asp or Cys in combination with nisin could ameliorate the potency of the lantibiotic against biofilms of *S. mutans* (Tong et al., 2014b). In an earlier study, the same group found that nisin interacted synergistically with sodium fluoride against *S. mutans* biofilms, in that the combination was more effective than sodium fluoride used independently at inhibiting the formation of biofilms at 4 and 16 h (Tong et al., 2011). The same investigators also assessed the effect of adding nisin to the dental irrigant, MTAD, and its anti-biofilm activity against oral *E. faecalis* isolates. Importantly, it was found that nisin in combination with doxycycline successfully inhibited *E. faecalis* biofilms whereas MTAD on its own was ineffective against such biofilms (Tong et al., 2013). *E. faecalis*, best known as a notorious nosocomial pathogen causing systemic infections, is also implicated in root canal infections. These successful combinations mentioned above could be harnessed as effective antimicrobial combinations against *E. faecalis* and *S. mutans* biofilms or as part of an endodontic irrigant following root canal treatments to prevent post-operative infections.

With respect to Gram negative organisms, a recent study found that nisin was effective when used together with polymyxins against *Pseudomonas aeruginosa* biofilms (Field et al., 2016b). *P. aeruginosa* is an important opportunistic pathogen and its biofilm-forming abilities contribute to its pathogenicity (Gellatly and Hancock, 2013). It is particularly common in lungs of patients with cystic fibrosis and thus warrants further extensive research to target its biofilm-forming and consequent pathogenic properties (Reen et al., 2016). The study by Field and co-workers demonstrated that decreased concentrations of polymyxins (either 0.5x MIC or even 0.2x MIC) are required to prevent *P. aeruginosa* PA-01 biofilm formation, when combined with 0.25x MIC nisin. Combinations of 0.33x MIC of nisin with 0.5x MIC of polymyxin were also effective against PA-01 planktonic cells (Field et al., 2016b; Figure 1A). Nisin was also used in combination with other antimicrobial agents used in the clinic against planktonic cells of *P. aeruginosa* ATCC 27853, as well as 40 other strains of *P. aeruginosa* in a separate study (Giacometti et al., 1999). A synergistic interaction of this nature can be hugely beneficial as it has the potential to attenuate the undesired nephrotoxicity associated with polymyxins.

A recent study using the sactibiotic, subtilosin, in combination with LAE was undertaken with a view to targeting *G. vaginalis* biofilms (Algburi et al., 2015). A combination of two methods; ATP viability assays and resazurin assays, were conducted to show the efficacy of these combinations against biofilms of the pathogen. Encouragingly, this combination of subtilosin-LAE was ineffective against healthy vaginal *Lactobacillus* biofilms. This highlights the specific nature of antimicrobial combinations and such a combination could be harnessed as a targeted therapeutic option against bacterial vaginosis. Furthermore, bacteriocins can potentially be combined with agents that target biofilms through non-microbiocidal means such as by altering molecular pathways which are responsible for regulating biofilm formation. Examples of such agents include thiazolidinone derivatives and diterpenoids (Buommino et al., 2014).

A summary of studies involving bacteriocins in combination with other stressors with a view to targeting clinical and veterinary pathogens, as well as biofilms, is found in Tables 1, 2.

**Table 1:** Bacteriocins in combination with other stressors against clinical and veterinary pathogens.

**Bacteriocin**	**Antimicrobial/Stressor**	**Target**	**Effect**	**References**
Nisin	Ramoplanin or chloramphenicol	MRSA	Synergy with ramoplanin against 14/20 strains; antagonism with chloramphenicol	Brumfitt et al., 2002
	Polymyxin E or clarithromycin	*P. aeruginosa*	Synergy	Giacometti et al., 1999
	Amoxicillin, penicillin, streptomycin ceftiofur, tetracycline	*S. suis*	Synergy with every combination	LeBel et al., 2013
	Chloramphenicol or penicillin	*E. faecalis*	Synergy with the antibiotics	Tong et al., 2014a
Actagardine	Ramoplanin	*C. difficile*	Partial synergy/additive against 8/13 strains	Mathur et al., 2013
	Metronidazole	*C. difficile*	Partial synergy/additive against 7/13 strains	Mathur et al., 2013
	Vancomycin	*C. difficile*	Partial synergy/additive against 5/13 strains	Mathur et al., 2013
Lacticin 3147	Polymyxin B or E	*E. faecium* DO	Indifference	Draper et al., 2013
	Polymyxin B	*S. aureus 5247*	Partial synergy	Draper et al., 2013
	Polymyxin E	*S. aureus 5247*	Indifference	Draper et al., 2013
Suicin 3908	Amoxicillin or penicillin	*S. suis*	Additive effects	Vaillancourt et al., 2015
Thuricin CD	Ramoplanin	*C. difficile*	Partial synergy/additive against 4/13 strains	Mathur et al., 2013
	Metronidazole	*C. difficile*	Indifference against 13/13 strains	Mathur et al., 2013
	Vancomycin	*C. difficile*	Partial synergy/additive against 2/13 strains	Mathur et al., 2013
Subtilosin A	Clindamycin phosphate or metronidazole	*G. vaginalis*	Synergy	Cavera et al., 2015
	Lauramide arginate or Ester poly-lysine	*G. vaginalis*	Bliss synergy	Cavera et al., 2015
Subtilosin	Lauric arginate, ϵ-poly-L-lysine, glycerol monolaurate	*G. vaginalis*	Synergy	Noll et al., 2012
PsVP-10	Triclosan	*S. mutans* and S. *sobrinus*	Partial Synergy	Lobos et al., 2009
	Chlorhexidine	*S. mutans* and S. *sobrinus*	Synergy	Lobos et al., 2009
Plantaricin E,F,J,K	Several antibiotics	*Candida albicans*	Synergy	Sharma and Srivastava, 2014
Durancin 61A	Reuterin	*C. difficile*	FIC values of 0.2 against *C. difficile*	Hanchi et al., 2017
	Vancomycin	MRSA	FIC values of 0.3 against *S. aureus* ATCC 700699	Hanchi et al., 2017

**Table 2:** Bacteriocins in combination with other antimicrobials/stressors against biofilms.

**Bacteriocin**	**Antimicrobial/Stressor**	**Target biofilm**	**Effect**	**References**
Nisin	Glu, Asp, Cys	*S. mutans*	Improved potency of nisin against biofilms	Tong et al., 2014b
	Sodium fluoride	*S. mutans*	Synergy	Tong et al., 2011
	Doxycycline	*E. faecalis*	Inhibition of biofilms	Tong et al., 2013
	Several antibiotics	MRSA ATCC43300	Synergy in preventing the formation of biofilms	Mataraci and Dosler, 2012
	Ciprofloxacin or daptomycin	MRSA	Decrease in MRSA CFU counts by 3 logs	Dosler and Mataraci, 2013
	Lysostaphin	*S. aureus*	Pre-formed biofilm reduced by >50% for 8 strains	Ceotto-Vigoder et al., 2016
	Polymyxin	*P. aeruginosa*	Reduced concentrations of polymyxins needed to inhibit biofilm formation	Field et al., 2016b
Nisin M21V	Penicillin	*S. aureus* SA113	Biofilm inhibited	Field et al., 2016a
Nisin I4V	Chloramphenicol	*S. pseudintermedius* DSM21284	Biofilm inhibited	Field et al., 2016a
Nisin M21A	Citric acid, cinnamaldehyde	*L. monocytogenes*	*L. monocytogenes* biofilm inhibited	Smith et al., 2016
Subtilosin	Lauramide arginine ethyl ester (LAE)	*G. vaginalis*	Effective at inhibiting biofilm	Algburi et al., 2015.
Enterocin AS-48	Triclosan, benzalkonium chloride, PHMG	MRSA	Effective when biocides were combined with 50 μg/ml AS-48	Caballero Gómez et al., 2013
Enterocin B3A-B3B	Nisin	*L. monocytogenes*	MIC needed to disrupt biofilm reduced	Al-Seraih et al., 2017

## Effects of antimicrobial combinations involving bacteriocins against food-borne pathogens

With regards to the food industry, disease-causing and spoilage organisms can have tremendous implications in terms of morbidity/mortality, as well as financial implications. Several persistent pathogens exist in food systems, both in planktonic states as well as in biofilms. Furthermore, there is an emphasis on attempting to replace chemically-derived antimicrobials in food with more natural antimicrobials such as nisin and plant-derived essential oils. The increase in the extent of global food distribution, in conjunction with more frequent travel has elicited an increase in the dissemination of food-borne diseases and solutions are required to combat this trend (Hussain and Dawson, 2013). While the use of chemical preservatives as well as heat treatment have proven to be successful in the past in limiting food-borne pathogens as part of the hurdle effect, such treatments can have an impact on the organoleptic properties of food. In addition, increasing pressure from consumers for safe to eat food which is minimally processed has ignited an interest in the development of effective natural antimicrobials or antimicrobial combinations to control food-borne pathogens.

### Combinations of bacteriocins with essential oils, naturally-derived compounds and preservatives against gram positive food-borne pathogens

The lantibiotic nisin has been the subject of several antimicrobial combination studies with a view to targeting food-borne pathogens. Indeed, it has been utilized for decades as a food preservative in more than 50 countries (Delves-Broughton, 1990). *L. monocytogenes*, the causative agent of listeriosis, is a notorious food-borne pathogen, and can cause opportunistic infections which can lead to meningitis and sepsis in severe cases (Low and Donachie, 1997; Schuppler and Loessner, 2010). In one study, it was found that 6,400 IU of nisin in combination with a green tea extract (GTE) or in combination with a grape seed extract (GSE) resulted in effective cell damage in a target *L. monocytogenes* strain (Sivarooban et al., 2008). The purified phenolic compounds present in GTE (0.02% epicatechin and 0.02% caffeic acid), as well as the pure phenolic compounds present in GSE (0.02% catechin and 0.02% epicatechin) were also used in the study (Sivarooban et al., 2008). When nisin was combined with GTE or GSE compounds, a compromised cell membrane and a condensed cytoplasm were apparent with TEM. With a starting inoculum of 10^6^ CFU/ml, the combination of nisin with GSE resulted in *Listeria* levels which were undetectable after 24 h of incubation, whereas the combination of nisin and GTE led to a decrease in *Listeria* levels to a mere 3.76 CFU/ml (Sivarooban et al., 2008). In another study, it was shown that semi-purified preparations of nisin A and a bioengineered derivative nisin V, combined effectively with the essential oils carvacrol, thymol and trans-cinnamaldehyde to inhibit *L. monocytogenes* in a validated food model system, as well as in laboratory media. It was shown that a greatly delayed lag phase was apparent during growth curves when nisin V was combined with 0.02% of the above-mentioned essential oils, compared to the nisin A equivalent (Field et al., 2015). Additionally, in time-kill assays, a 2-log decrease in cell numbers over and above that achieved by the nisin A combination with carvacrol or cinnamaldehyde was observed against the target *L. monocytogenes* EGDe, which was also maintained in a number of food settings (Field et al., 2015). In a separate study, the antimicrobial activity of nisin Z was potentiated when combined with thymol at sub-inhibitory concentrations, against the target strain *L. monocytogenes* ATCC7644 (Ettayebi et al., 2000). Significantly, the study showed that 40 IU/ml nisin combined with 0.02% thymol was more effective at inhibiting ATCC7644 than either of the antimicrobials used on their own. A combination of this nature may also preclude the emergence of bacterial sub-populations displaying resistance to the lantibiotic (Ettayebi et al., 2000). Another study investigated the effects of combining nisin with carvacrol or mountain savory essential oils on the viability of target *L. monocytogenes* strains subsequent to γ-irradiation. There was an increase in the relative sensitivity of *L. monocytogenes* to γ-irradation by a factor of 6.31 when nisin and mountain savory essential oils were combined and a corresponding increase in sensitivity by a factor of 4.19 with nisin-carvacrol combinations (Ndoti-Nembe et al., 2013).

With regards to combinations of bacteriocins with preservatives, a study reported that combinations of nisin-potassium sorbate-ethylene diamine tetraacetic acid (EDTA), as well as combinations of nisin-sodium-diacetate-EDTA were effective at reducing the numbers of *L. monocytogenes* on shrimp inoculated with the pathogen (Wan Norhana et al., 2012). Synergistic interactions between nisin and low concentrations of EDTA in targeting *L. monocytogenes* were also described in a study by Branen and Davidson. In addition, the authors showed that the antimicrobial activity of nisin against *L. monocytogenes* was potentiated in the presence of lactoferrin in the same study (Branen and Davidson, 2004). Combinations of 10 IU/ml nisin with 250 μg/ml lactoferrin were also shown to perform synergistically to inhibit *L. monocytogenes* in a separate study, similar to Branen and Davidson's findings (Murdock et al., 2007).

With respect to other classes of bacteriocins, Grande and co-workers reported synergistic effects when the class IIc bacteriocin enterocin AS-48 was combined with the phenolic compound carvacrol (Grande et al., 2007). The authors conducted a food trial with vegetable sauces whereby they investigated the efficacy of AS-48 both independently and when combined with phenolic substances with a view to inhibiting *S. aureus*. The activity of AS-48 was significantly enhanced against *S. aureus*, when combined with eugenol, geraniol, terpineol, carvacrol, hydrocinnamic acid, caffeic acid, citral and p-coumaric acid. The levels of the pathogen were decreased to less than detectable levels when 80 μg/ml of AS-48 was mixed with 126 nM carvacrol or 20 mM hydrocinnamic acid, in carbonara sauce kept at 22°C. Overall, the degree of synergy obtained depended on the concentration of the antimicrobials and also the type of sauce used (Grande et al., 2007). Enterocin AS-48 was also shown to interact in a synergistic manner with lactic acid, p-hydroxybenzoic methylester acid (PHBME) and citric acid in a separate study (Antonio et al., 2009). In salads spiked with *L. monocytogenes*, the application of 30 μg/g of AS-48 in combination with PHBME, Nisaplin or lactic acid led to a significant reduction in *L. monocytogenes* numbers (Antonio et al., 2009). Amrouche and co-workers showed that the sactibiotic bacteriocin subtlosin was effective at inhibiting *L. monocytogenes* Scott A and NR30 when combined with ε-poly-L-lysine, zinc lactate or with curcumin (Amrouche et al., 2010). The strain *L. monocytogenes* Scott A was originally implicated in a listeriosis outbreak in Massachusetts in 1983 (Fleming et al., 1985; Briers et al., 2011) while the strain NR-30 has been reported to display resistance to nisin (Mazzotta and Montville, 1997). Partial synergy against *L. monocytogenes* Scott A was apparent when subtilosin was used in conjunction with an encapsulated form of curcumin in the study by Amrouche et al., while subtilosin-zinc lactate combinations also exhibited synergy against both NR30 and Scott A. However, when combined with non-encapsulated poly-L-lysine or curcumin, subtilosin displayed merely additive effects against the two *L. monocytogenes* strains (Amrouche et al., 2010). Finally, a separate study reported that the combination of a cell-bound bacteriocin produced by *Lactobacillus curvatus* CWBI-B28 with savory essential oil or oregano was effective at reducing *L. monocytogenes* to below detectable levels in pork meat spiked with the pathogen, after a week of storage (Ghalfi et al., 2007). The oregano essential oil combined with the cell-bound bacteriocin was still effective after 3 weeks of storage, whereas all other combinations led to an increase in *L. monocytogenes* levels after the same duration of time. Overall, this cell-bound bacteriocin combined with savory essential oil was found to retard the growth of *L. monocytogenes* by 2 weeks, relative to the use of the cell-bound bacteriocin on its own (Ghalfi et al., 2007).

*L. monocytogenes* has the ability to form biofilms in addition to growing in a planktonic state, and a recent study demonstrated the effectiveness of the bioengineered derivative of nisin, M21A, in combination with natural food-grade additives (cinnamaldehyde and citric acid) in targeting biofilms of strain F6854 (Smith et al., 2016). This strain has been associated with contaminated turkey frankfurters (Nelson et al., 2004). The study by Smith et al., showed that the bioengineered nisin derivative M21A (0.1 μg/ml) was more effective at inhibiting F6954 biofilms than wild-type nisin, when combined with citric acid (175 μg/ml) or cinnamaldehyde (35 μg/ml) (Smith et al., 2016). A separate study recently reported that the class IIb bacteriocin, enterocin B3A-B3B, was effective when used in combination with nisin at decreasing the MIC needed to disrupt the growth of *L. monocytogenes* in either a planktonic state or when present as a biofilm (Al-Seraih et al., 2017). Finally, the cell-free supernatant (CFS) containing a bacteriocin from *Lb. curvatus* ET31 was tested in combination with EDTA and the investigators noted that while the bacteriocin and the EDTA independently were ineffective against biofilms which had already formed, the CFS combined with EDTA was effective at reducing the viability of *L. monocytogenes* biofilms which had already formed, whilst not fully eliminating the biofilms (Camargo et al., 2016).

### Bacteriocins in combination with other stressors against gram negative food-borne pathogens

Gram negative pathogens in particular have proven to be more problematic to tackle using bacteriocins than Gram positive pathogens primarily because of the outer membrane present in Gram negatives that limits access to the cell membrane. Importantly however, the bioengineered nisin variants S29A and S29G have been shown to display activity against Gram negatives (Field et al., 2012). While nisin A has been shown to be effective against Gram negatives when used in combination with chelating agents such as EDTA, perhaps a more attractive option is combining nisin with natural phenolic compounds such as thymol and carvacrol, which facilitate the permeabilization/disruption of the membrane (Stevens et al., 1991; Helander et al., 1998). However, as concentrations of essential oils which are needed to have antimicrobial activity can compromise the organoleptic qualities of foods, their use as preservatives in high concentrations have been rather limited thus far.

Nonetheless, a recent study utilized nisin A, and its bioengineered derivatives nisin S29A and nisin M21V independently and in combination with the essential oils carvacrol, trans-cinnamaldehyde, thymol, as well as the preservative citric acid, to evaluate the efficacy of such combinations against the Gram negative food-borne pathogens *Escherichia coli* O157: H7 and *Cronobacter sakazakii* (Campion et al., 2017). *E. coli* O157: H7 is an enterohaemorrhagic strain and has been associated with outbreaks in the US, Canada, UK and Japan (Besser et al., 1993; Bach et al., 2002; Vidovic and Korber, 2016). *C. sakazakii* has been associated with contaminated infant milk formula and is implicated in cases of enteritis, meningitis and septicaemia (Gurtler et al., 2005; Drudy et al., 2006; Iversen and Forsythe, 2007; Yan et al., 2012). Campion et al., noted that extended lag phases of *C. sakazakii* and O157: H7 were apparent when 30 μM of the bioengineered nisin variants were combined with 0.035% trans-cinnamaldehyde, 0.03% carvacrol and 0.015% thymol, when compared to corresponding combinations of nisin A-essential oils. In addition, a 4-log reduction of *C. sakazakii* and a 3-log reduction in viable counts of O157: H7 was particular noteworthy when 60 μM of the nisin variants were combined with 0.03% carvacrol, in comparison to corresponding nisin A-carvacrol combinations (Figure 1D). Importantly, the study also showed that when stored at room temperature, sub-lethal concentrations of nisin variants in combination with carvacrol were successful in fully inactivating O157: H7 in apple juice, again when compared to nisin A-carvacrol combinations. Similarly, the commercial product Nisaplin at concentrations of 10 mg/ml, in combination with 30 mM citric acid, elicited >3 log decreases in *C. sakazakii* viable counts in infant formula after 3 h of incubation (Campion et al., 2017). The increased stability of nisin at lower pH conditions compounded by the ameliorated diffusion properties of essential oils in acidic conditions could explain the effective combinations in low-pH drinks such as apple juice (Delves-Broughton, 1990; Burt, 2004; Campion et al., 2017). As thymol and carvacrol disrupt Gram negative outer membranes (which renders them more sensitive to nisin), while trans-cinnamaldehyde disrupts the transmembrane ATPase, combinations of such oils with nisin may prove to be the most effective option to control Gram negative food-borne pathogens (Helander et al., 1998; Gill and Holley, 2006a,b).

With regards to other studies investigating bacteriocin synergy against Gram negative food-borne pathogens, Moon et al., reported that a bacteriocin 4.5 kDa in size from *Pediococcus acidilactici* K10 in combination with the organic acids lactic acid, succinic acid and acetic acid interacted synergistically against *E. coli* O157: H7 both *in vitro* and *in situ*. This bacteriocin from *P. acidilactici* K10 in combination with 0.35% lactic acid or 0.25% acetic acid was evaluated in a ground beef sample at 4°C and it was noteworthy that a 2.8-log reduction of O157: H7 was observed with lactic acid combinations (Moon et al., 2002). Thus, *P. acidilactici* and organic acid combinations may have potential as food bio-preservatives. Branen and Davidson showed the efficacy of nisin combined with EDTA against enterohaemorrhagic *E. coli* strains (Branen and Davidson, 2004) while a separate study demonstrated the efficacy of 250 IU/ml of nisin combined with 500 μg/ml lactoferrin at preventing *E. coli* O157: H7 growth (Murdock et al., 2007). Finally, Ananou *et al.*, observed synergistic effects when enterocin AS-48 was combined with agents that disrupted the outer membrane of a pathogenic *E. coli* O157: H7 isolate (Ananou et al., 2005).

A summary of studies involving bacteriocins in combination with various stressors with a view to targeting food-borne pathogens is found in Table 3.

**Table 3:** Bacteriocins in combination with naturally-derived compounds against food-borne pathogens.

**Bacteriocin**	**Antimicrobial/Stressor**	**Target**	**Effect**	**Reference**
Nisin	Green tea extract or grape seed extract	*L. monocytogenes*	Decrease in *Listeria* levels, compromised cell membrane and condensed cytoplasm	Sivarooban et al., 2008
	Carvacrol or mountain savory essential oils	*L. monocytogenes*	Increased sensitivity to γ-irradiation	Ndoti-Nembe et al., 2013
	Cefotaxime or ceftriaxone	*Salmonella* Typhimurium	Synergy	Singh et al., 2013
	EDTA	*Salmonella* Typhimurium	Additive	Singh et al., 2013
	Pediocin PA1	*E. coli, L. monocytogenes*	Synergy against *L. monocytogenes*; ineffective against *E. coli*	Naghmouchi et al., 2011
Nisin Z	Thymol	*L. monocytogenes* and *B. cereus*	Dose of nisin Z required reduced	Ettayebi et al., 2000
Nisin V	Carvacrol, trans-cinnamadehyde or thymol	*L. monocytogenes*	Extended log phase	Field et al., 2015
Nisin S29A or M21V	Carvacrol, trans-cinnamadehyde or thymol	*E. coli* O157: H7 and *C. sakazakii*	Extended lag phase of strains. Viable counts of strains decreased with carvacrol combinations. Also, O157: H7 inactivated in apple juice trial with carvacrol combinations	Campion et al., 2017
Subtilosin	Encapsulated curcumin	*L. monocytogenes* Scott A	Partial synergy	Amrouche et al., 2010
	Zinc lactate	*L. monocytogenes* Scott A and NR30	Synergy	Amrouche et al., 2010
	Non-encapsulated ε-poly-L-lysine, curcumin	*L. monocytogenes* Scott A and NR30	Additive	Amrouche et al., 2010
Enterocin AS-48	Carvacrol or hydrocinnamic acid	*S. aureus*	Synergy	Grande et al., 2007
	Eugenol, geraniol, terpineol, carvacrol, hydrocinnamic acid, caffeic acid, citral and p-coumaric acid	*S. aureus*	Enhanced activity of enterocin As-48	Grande et al., 2007
	Lactic acid, PHBME or citric acid	*L. monocytogenes*	Synergy	Antonio et al., 2009
Pediocin PA1	Polymyxin E	*L. monocytogenes*	Reduced growth of *L. monocytogenes* in log and stationary phases	Naghmouchi et al., 2011
Bacteriocin from *Lb. curvatus* CWBI-B28	Savory essential oil, oregano	*L. monocytogenes*	Growth of *L. monocytogenes* retarded by 2 weeks	Ghalfi et al., 2007
Bacteriocin from *P. acidilactici* K10	Lactic acid, succinic acid, aceric acid	*E. coli* O157: H7	Synergy *in vitro* and *in situ*	Moon et al., 2002

### Bacteriocins in combination with antibiotics against food-borne pathogens

While antibiotics are unlikely to be used in foods, several studies have nonetheless investigated their efficacies in combination with bacteriocins against food-borne pathogens in laboratory conditions. Studies of this nature can be useful with regards to providing insights into the mechanisms of synergistic interactions, especially when bacteriocins are combined with antibiotics with known modes of action. *Bacillus cereus* is an important food-borne pathogen and several strains have the ability to cause food poisoning, often resulting in vomiting and diarrhea (Schoeni and Wong, 2005). In a relatively recent study, it was shown that the two-component lantibiotic, lacticin 3147, interacted synergistically with the antibiotic polymyxin B against *B. cereus* 8079 and *B. cereus* 5247 (Draper et al., 2013). Interestingly however, lacticin 3147-polymyxin E combinations resulted in indifferent (1.0 < FIC < 2.0) and antagonistic effects (FIC > 2.0), in contrast to the above-mentioned synergistic interactions with polymyxin B against the same targets, showing that a single amino acid change in the polymyxin backbone can lead to profound differences in terms of interactions with lacticin 3147 against specific target strains. The lantibiotic nisin and class IIa bacteriocin, pediocin PA-1, have also been combined with the antibiotic polymyxin E to target *L. monocytogenes* and *E. coli* isolates which had exhibited resistance to pediocin and polymyxin E respectively (Naghmouchi et al., 2011). Polymyxin and nisin combinations at concentrations of 0.6 μg/ml and 15.6 μg/ml; 4.7 μg/ml and 62.5 μg/ml; and 9.3 μg/ml and 32 μg/ml, respectively, resulted in the inhibition of polymyxin-resistant *E. coli*, pediocin PA-1-resistant *L. monocytogenes* and nisin-resistant *L. monocytogenes* variants by 74, 97, and 94% respectively, relative to the controls. While nisin A and pediocin PA-1 combinations were synergistic against *L. monocytogenes* and its resistant variants, they were ineffective against *E. coli* or its resistant variants. Nonetheless, nisin-polymyxin combinations at concentrations of 7.8 μg/ml and 0.3 μg/ml, respectively, and polymyxin independently at a concentration of 0.21 μg/ml decreased the growth of log phase *E. coli* cells by approximately 94 and 31% respectively. Pediocin PA-1-polymyxin E combinations at concentrations of 25 μg/ml and 4.7 μg/ml, respectively, reduced the growth of *L. monocytogenes* in the exponential and stationary phases by 90% and 78%, respectively. Thus, the study indicated that resistant *L. monocytogenes* and *E. coli* isolates can be managed by using combinations of nisin/polymyxin E or pediocin PA-1/polymyxin E respectively (Naghmouchi et al., 2011). The same group also evaluated the activity of colistin in combination with pediocin PA-1/AcH or nisin with a view to targeting *E. coli* O157: H7, *Yersinia enterocolitica* ATCC 9610, *P. aeruginosa* ATCC 27853 and *Salmonella choleraesuis* ATCC 14028. Significantly, 1.56 μg/ml of pediocin PA-1/AcH or 1.7 μg/ml of nisin in combination with colistin elicited a marked reduction in the concentration of colistin needed to inhibit O157: H7 (Naghmouchi et al., 2013).

With respect to studies involving other Gram negative food-borne pathogens, lacticin 3147 was reported to exhibit synergy when combined with polymyxin B against *C. sakazakii* DPC6440 with FIC values of 0.25 (Draper et al., 2013). The lantibiotic also exhibited synergistic activity against the same strain when combined with polymyxin E with corresponding FIC values of 0.062 against DPC6440. Interestingly however, indifferent and antagonistic effects were obtained when lacticin 3147 was combined with polymyxin B or polymyxin E against the target strains *Salmonella* Typhimurium UK1 and LT2, with FIC values greater than 1.125 in all cases (Draper et al., 2013). A separate study by Rishi and co-workers demonstrated the efficacy of β-lactam antibiotics combined with nisin with a view to inhibiting the food-borne pathogen *Salmonella enterica* serovar *Typhi* (Rishi et al., 2014). Nisin-β-lactam synergistic combinations were assessed by conducting FIC and time-kill assays and with the exception of three strains, synergy was observed with all combinations against the clinical *Salmonella* strains in the *in vitro* study by Rishi and co-workers with nisin-cefotaxime and nisin-ceftriaxone proving to be the most effective combinations (Rishi et al., 2014). Singh et al., in a similar study, also evaluated the effectiveness of nisin in combination with standard antibiotics against multi-drug resistant strains of *Salmonella* and any such synergistic interactions were evaluated by FIC determinations using the checkerboard assay as well as time-kill assays and radial diffusion assays (Singh et al., 2013). Furthermore, scanning electron microscopy (SEM) and mouse trials assessing the combinatorial interactions were also conducted to validate synergistic effects observed with *in vitro* assays. Decreases in the numbers of *Salmonella* in various organs of infected mice were observed as a consequence of the antimicrobial combination. Using FIC values and time-kill assays, nisin-cefotaxime and nisin-ceftriaxone combinations yielded synergistic effects whereas nisin-EDTA and nisin-ampicillin combinations yielded additive effects against serovar Typhimurium. Significant alterations in the outer membrane of the target cells, elicited by the antimicrobial combinations were apparent and bacteriocin-β-lactam combinations caused greater log decreases of *Salmonella* in the spleen, intestine and liver of mice, relative to treatment with the antimicrobials independently (Singh et al., 2013). More specifically, the combination of nisin (at concentrations of 25 mg/Kg body weight and 50 mg/Kg body weight) with ceftriaxone (also at concentrations of 25 mg/Kg and 50 mg/Kg) resulted in 2.83, 3.11, 2.6, and 3.1-log decreases in *Salmonella* respectively in the spleen. Identical concentrations of nisin and cefotaxime were also combined, resulting in 2.06, 2.49, 2.11, and 2.44-log decreases respectively in the spleen as well. In contrast, 25 mg/Kg nisin and 50 mg/Kg nisin administered independently resulted in a mere 0.16 and 0.3-log decrease respectively in *Salmonella* in the spleen. Similarly, combinations of nisin (25 mg/Kg body weight and 50 mg/Kg body weight) combined with identical concentrations of ceftriaxone proved to be effective, resulting in decreases in *Salmonella* in the liver ranging from 2.75 to 3.5-log units. Corresponding combinations of nisin and cefotaxime resulted in reductions in *Salmonella* in the liver ranging from 2.27 to 3.26-log units. These values were higher than the 0.42 to 0.67-log unit reductions achieved by nisin independently. Following on from this study, the same group attempted to elucidate the mechanism of these synergistic interactions (Singh et al., 2014). Essentially, the ability of nisin and the β-lactams to target the cell membrane was evaluated by conducting membrane permeabilizing assays in combination with pulse labeling techniques. The results showed that the bacteriocin-β-lactam combination affected membrane permeability, as confirmed by the uptake of 1-N-phenylnapthylamine (NPN) by the treated cells. This uptake of NPN as a consequence of membrane permeabilization, as well as interference with DNA, RNA and protein synthesis was dependent on both the dose of the antimicrobials, as well as the duration of exposure to the antimicrobials in combination. Significantly, results of *in vivo* assays involving mouse trials corroborated synergistic effects seen in *in vitro* assays in the study (Singh et al., 2014). Thus, nisin-cefotaxime and nisin-ceftriaxone synergistic effects against *Salmonella* were predominantly due to permeabilization of the membrane, as well as DNA, RNA, protein synthesis inhibition, and immune-modulatory activity (Singh et al., 2014).

## Other types of effective interactions against pathogens involving bacteriocins

A potential strategy with a view to combating recalcitrance to traditional antibiotics may be to combine bacteriocins with phages/endolysins and some studies have already investigated such prospects. For instance, a recent study evaluated the efficacy of combinations of the class II bacteriocin coagulin C23 with listeriaphages against *L. monocytogenes* and found that they act in a synergistic manner against the food-borne pathogen (Rodríguez-Rubio et al., 2015). More specifically, coagulin C23 was combined with the phages FWLLm1 or FWLLm3 and synergistic effects were apparent when the two antimicrobials were mixed in sub-inhibitory concentrations. Encouragingly, *L. monocytogenes* 2000/47 levels were lower than 10 CFU/ml after 96 h of storage at 4°C, when the bacteriocin was combined with the phage FWLLm1. However, the combination of coagulin C23 and FWLLm3 was not effective at inhibiting *L. monocytogenes* 2000/47 and this could be attributed to the emergence of mutants resistant to coagulin C23 and FWLLm3. Significantly, the authors in the study concluded that the rate of development of resistance was higher when the antimicrobials were used independently, relative to the combination of the two antimicrobials together. This phenomenon of delayed resistance development could explain the synergistic effects observed in the study (Rodríguez-Rubio et al., 2015).

Synergy was also observed when nisin was combined with the *S. aureus* lytic phages phiϕ35 and ϕ88 in another study (Martínez et al., 2008). Unfortunately, the use of this combination as a viable therapeutic option has been hindered due to the emergence of resistance to the two phages employed and adaptation to the lantibiotic nisin. With regards to other studies relating to phages combined with bacteriocin-like agents with a view to targeting *S. aureus*, the bacteriolysin lysostaphin exhibited synergistic effects in combination with the two endolysins (LysK) against MRSA (Dajcs et al., 2002; O'Flaherty et al., 2005; Becker et al., 2008). A deeper understanding of the mechanisms of synergistic interactions between bacteriocins and endolysins is essential if such an interaction is to be used in food/clinical settings. In the case of lysostaphin-LysK synergy, it may be that LysK by virtue of the fact that it has two lytic domains, has the ability to further potentiate the lytic nature of lysostaphin, which merely possesses one lytic domain (Becker et al., 2008). Finally, a study by Garcia et al., also reported synergistic interactions between nisin and the phage endolysin LysH5 (García et al., 2010). The activity of LysH5 may be enhanced by the ability of nisin to permeabilize the cell membrane of target *S. aureus* strains (Nascimento et al., 2008; Obeso et al., 2008).

## The use of mathematical models to assess antimicrobial synergy and predict resistance development

While synergistic antimicrobial interactions are likely to be beneficial in clinical applications, the emergence of multi-drug resistance arising from such interactions remains unclear. Recently, mathematical modeling has enhanced the predictive capabilities of such antimicrobial interactions with regards to development of resistance (Ankomah and Levin, 2012; Chen et al., 2015). Indeed, Torella et al., addressed this phenomenon by optimizing a mathematical model to study infections *in vivo* and found that there were two opposite effects of synergy: (i) the synergistic interaction results in more rapid clearance of the infection and consequently fewer opportunities for the development of resistant derivatives/mutants and (ii) the selection of resistant isolates/derivatives is favored over wild-type cells associated with synergistic interactions (Torella et al., 2010). The authors found that when resources are abundant, the synergistic effects are more potent at eliminating the infection but conversely, when resources are limited, the potential for development of multi-drug resistance also increases. Above a certain critical level of drug interaction, the potential for emergence of multi-drug resistance is enhanced. Interestingly, the study suggested that to dampen down the emergence of multi-drug resistance, antimicrobial antagonism may on occasions actually be better than antimicrobial synergy (Torella et al., 2010).

In a similar study, Landersdorfer et al. devised a model for evaluating the synergistic interactions of antibiotic combinations using a sequential dosing design (Landersdorfer et al., 2013). The authors used nisin in combination with either linezolid or amikacin for the study. Sequential, as well as simultaneous, administration of the antimicrobials enabled the evaluation of the efficacy of linezolid or amikacin against populations of cells which were nisin-resistant or nisin-intermediate cells. Landersdorfer et al. used the software NONMEM and S-ADAPT to model the synergistic interactions. The study found that while bacterial replication was inhibited by linezolid in populations less sensitive to nisin, this population of cells was not efficiently killed. The combination of amikacin with the lantibiotic nisin resulted in sub-population synergy. Such models of simultaneous or sequential antimicrobial dosing models may enable scientists to devise effective antimicrobial combination strategies for clinical applications (Landersdorfer et al., 2013).

In a landmark study, the concept of “the smile-frown transition” with respect to antimicrobial synergy was introduced (Pena-Miller et al., 2013). The authors used mathematical modeling, whole genome sequencing, genetic manipulation of resistance mechanisms and evolution experiments to demonstrate that synergistic antimicrobial combinations can be ineffective unless the first round of treatment results in bacterial clearance. It was suggested that the potency of antimicrobial synergy decreases concurrently with the emergence of drug-resistant bacteria. Evolution experiments exhibited that the efficacy of the antibiotics used exponentially decreased over a 5 day period. The authors also found that the replication of drug-resistant bacteria was fastest when the drug-sensitive counterparts were killed by aggressive treatment strategies (Pena-Miller et al., 2013). The initial synergistic interaction created a selective pressure for the emergence of resistance causing antagonistic effects after day 1 of the 5-day experiment. The authors dubbed this phenomenon “the smile-frown transition.” Genome sequencing in the study showed that emergence of resistance to the antibiotics may be due to the amplification of genes involved in drug-resistance mechanisms such as the *acrAB* efflux operon (Pena-Miller et al., 2013). The deletion of this *acrAB* operon precluded the transition from antimicrobial synergy to antimicrobial antagonism within 5 days of the evolution experiments. Thus, evidence such as that outlined by Pena-Miller implies that super-inhibitory concentrations of the two antimicrobials in combination may need to be used until the pathogen is fully cleared. Conversely, in the presence of sub-inhibitory concentrations of the two antimicrobials, the “smile-frown transition” is likely to take precedence (Pena-Miller et al., 2013).

## Conclusions

The 20th century was the golden era for the discovery of novel antibiotics and successful infection control strategies. However, the over-prescription of broad-spectrum antibiotics by clinicians worldwide in combination with overuse in animal applications has triggered an increase in antibiotic resistance and, in addition, has contributed to nosocomial infections such as *C. difficile* infection (CDI), due to perturbations of the gut microbiota. Furthermore, the broad-spectrum nature of several antibiotics as well as the negative links associated with the causation of autoimmune and atopic diseases with certain antibiotics renders them unattractive options (Blaser, 2011; Willing et al., 2011). The over-exposure to antibiotics in the environment, healthcare settings and in agriculture has contributed to this problem. Furthermore, the increase in global travel, compounded by poor infection control standards, has also exacerbated the crisis (Holmes et al., 2016). In particular, in hospital settings, the dissemination of antibiotic-resistant pathogens, especially in immunosuppressed patients is a cause for concern. A thorough understanding of the mechanisms of antibiotic resistance is urgently warranted to mitigate this global concern. Alternative therapeutic options, including bacteriocins used either independently or in combination with other stressors must also be thoroughly explored. Amongst the key advantages of bacteriocins include their ribosomally-synthesized nature, which renders them amenable to bioengineering strategies. Such bioengineered variants may possess enhanced bioactivity against certain clinical/food-borne pathogens or food spoilage organisms, as well as potentially possessing ameliorated physicochemical properties such as improved solubility, protease resistance and pH tolerance, further augmenting their value and effectiveness as antimicrobials. Other potential advantages of using bacteriocins include their high potency against target strains, their stability and their low toxicity. A potential disadvantage is that oral ingestion is complicated due to their proteolytic digestion in the gut. However, this may be overcome by advances in encapsulation technologies. Another means to circumvent proteolytic breakdown is to administer them parenterally for systemic applications.

Although there have already been studies conducted which have revealed potentially promising synergistic interactions between bacteriocins and other stressors, it must be highlighted that, since there are a large number of bacteriocin-antimicrobial combinations that have yet to be investigated, there could still be very useful combinations against targeted pathogens which are currently untapped. Thus, far, a precise understanding of the mechanism of synergistic interactions of antimicrobial combinations has hindered the progress of alternative therapeutic options of bacteriocin-antimicrobial combinations against target strains, particularly in clinical settings. Indeed, there has been a general reluctance in resorting to alternative therapeutic options and changing the status quo in the clinical arena. Elucidation of the mode of action of these synergistic interactions using a combination of genomic, transcriptomic and proteomic tools is likely to expedite the processes involved in the deployment of these antimicrobial combinations in clinical and/or food settings.

With respect to the clinical efficacy of bacteriocin-antimicrobial combinations, the precise nature of physicochemical interactions, such as hydrophobic-hydrophobic or cationic-anionic interactions, between a proteinaceous bacteriocin and an antibiotic are likely to be important considerations when optimizing effective combinatorial therapy for use *in vivo*. In this regard, it may also be the case that combinations of two bacteriocins that are of a similar molecular weight may be more effective *in vivo* than combining a high molecular weight bacteriocin with a low molecular weight antibiotic. In addition, the pharmacodynamic and pharmacokinetic traits of a bacteriocin are likely to be critical factors determining its success as a potential therapeutic agent *in vivo*. This becomes even more relevant when combined with antibiotics as physicochemical interactions of the bacteriocin with the antibiotic can interfere with the pharmacodynamic properties of both antimicrobials. With respect to the pharmacokinetic properties of bacteriocins in combination with antibiotics, optimization of the route of administration of the two antimicrobials to the localized site of infection is likely to be an important step in determining the success of the treatment. Indeed, localized cutaneous, intravaginal or inhaled routes of administration of bacteriocins may be effective due to the relatively low absorption rates, minimizing potentially undesired systemic side effects (Ghobrial et al., 2009, 2010a; van Heel et al., 2011). However, this may be complicated in the presence of an antibiotic used in combination. With respect to systemic applications however, the lantibiotic group of bacteriocins in particular could prove to be less efficacious due to their propensity to bind blood components (Ghobrial et al., 2010b). Thus, the distribution and consequent bioavailability of such bacteriocins in the desired target site can unfortunately be significantly attenuated. Aside from reducing the bioavailability, the binding of bacteriocins to plasma proteins can also reduce the specific activity of the bacteriocin against a target strain by potentially hindering access of the bacteriocin to its target receptor (Ghobrial et al., 2010b). This problem can be further exacerbated due to the instability of certain lantibiotics under physiological pH conditions. However, bioengineering strategies with a view to seeking derivatives with enhanced stability has the potential to somewhat mitigate this issue (Rollema et al., 1995; Yuan et al., 2004). It must also be highlighted that differences in the rates of metabolism and excretion of bacteriocins combined with antibiotics are likely to exist between animals and humans, and the half-life of each of the antimicrobials can have an impact on the propensity for development of resistance. In addition, further insights with respect to the effects of such antimicrobial combinations on eukaryotic cells are also essential in order to prevent any undesired side effects. Encouragingly, the evidence accumulated thus far has shown that bacteriocins in general tend to display low toxicity rates against epithelial cells and with the exception of cytolysin, generally tend to exhibit extremely low levels of hemolysis (Cox et al., 2005; Maher and McClean, 2006; Aranha et al., 2008). However, all these factors above can be further complicated and clinical outcomes difficult to predict when bacteriocins are combined with other antimicrobials. Overall, optimization of effective therapeutic concentrations of bacteriocins, either independently or in combination with other antimicrobials can only truly be achieved with the availability of more data concerning the pharmacokinetic properties of each of the antimicrobials in question. Therefore, a complex interplay of factors is bound to be crucial in governing the clinical efficacy of such potential combinatorial therapeutic options.

While bacteriocins interacting synergistically with other antimicrobials and stressors could have great potential in clinical and food settings, one must be aware that unfortunately, bacteriocins are by no means a “magic bullet” and are not exempt from development of resistance (Modi et al., 2000; Draper et al., 2015). Indeed, several different mechanisms of resistance to the lantibiotic subclass of bacteriocins have been described, which include cell-envelope altering mechanisms utilized by bacteria such as DltA or MprF (Peschel et al., 1999; Poyart et al., 2001; Abachin et al., 2002; Kovacs et al., 2006; Khattar et al., 2009; McBride and Sonenshein, 2011a,b), two component systems such as CprK in *C. difficile* (McBride and Sonenshein, 2011a,b; Suarez et al., 2013) and LisRK in *L. monocytogenes* (Cotter et al., 1999; Kallipolitis and Ingmer, 2001), as well as other mechanisms such as production of nisin resistance proteins (Chatterjee et al., 2005; O'Driscoll et al., 2006; Khosa et al., 2013) (for a comprehensive review on lantibiotic resistance, see Draper et al., 2015). Resistance to the class II group of bacteriocins have also been described in laboratory conditions and is likely to be mediated through decreased expression of Man-PTS receptors (Kjos et al., 2011). Ultimately, greater insights into the precise mechanisms of development of resistance to bacteriocins will facilitate their deployment in both clinical settings and as preservatives in food, either individually or in combination with other antimicrobials.

While it is plausible that the combination of two antimicrobials with two distinct modes of action attenuates the likelihood of resistance development, the emergence of sub-populations of target pathogens recalcitrant to both the bacteriocin as well as the other antimicrobial used in combination, remains a very realistic worst case scenario. It is interesting, in this regard, to note that the development of resistance to bacteriocins over the years has generally been associated with a slower growth rate and an associated fitness cost in resistant variants. Even though bacteriocins have been studied for several decades, the precise mechanisms of antimicrobial action of several of these peptides are still unknown. Knowledge of the exact modes of action of such bacteriocins would potentially help researchers to tailor-make “designer bacteriocins” which may act synergistically with other antimicrobials, with a view to targeting specific pathogens. By doing so, researchers could combine bacteriocins with certain antimicrobials whose modes of action are already known, in a target-specific manner. Finally, it must be highlighted that successful synergistic interactions between bacteriocins and other antimicrobials *in vitro* may not necessarily correlate with clinical efficacy. Nonetheless, optimization of a variety of complex factors including the pharmacodynamics/pharmacokinetic properties of the antimicrobials as well as antimicrobial concentrations and ratios at which the antimicrobials work in a synergistic fashion can lead to effective alternative therapeutic options with the ultimate view to confronting the increasingly worrying problem of antibiotic resistance.

## Author contributions

HM, DF, MR, PC, CH and RP wrote the manuscript. All authors read and approved the final manuscript.

### Conflict of interest statement

The authors declare that the research was conducted in the absence of any commercial or financial relationships that could be construed as a potential conflict of interest.

## Abbreviations

MRSA: methicillin-resistant *Staphylococcus aureus*
VRE: vancomycin-resistant enterococci
Man-PTS: mannose phosphotransferase
FIC: fractional inhibitory concentration
MCBT: multiple combination bactericidal test
PBP: penicillin binding protein
MIC: minimum inhibitory concentration
MBC: minimum bactericidal concentration
TEM: transmission electron microscopy
CDAD: *Clostridium difficile*-associated diarrhea
SAM: S-adenosylmethionine
LAE: lauramide arginine ethyl ester
ATCC: American Type Culture Collection
XTT: 2,3-Bis-(2-methoxy-4-nitro-5-sulfophenyl)-2H-tetrazolium-5-carboxanilide
MSSA: methicillin-sensitive *Staphylococcus aureus*
CFU: colony forming units
CLSM: confocal laser scanning microscopy
PHMG: polyhexamethylene guanidinium chloride
MTAD: mixture of tetracycline isonomer, acid and detergent
ATP: adenosine triphosphate
GTE: green tea extract
GSE: grape seed extract
EDTA: ethylene diamine tetraacetic acid
PHBME: p-hydroxybenzoic methylester acid
CFS: cell-free supernatants
DPC: Dairy Products Culture Collection
SEM: scanning electron microscopy
NPN: 1-N-phenylnapthylamine
DNA: deoxyribonucleic acid
RNA: ribonucleic acid
NONMEM: non-linear mixed effects modeling tool
CDI: *C. difficile* infection.
